# Macrophage Inducible C-Type Lectin As a Multifunctional Player in Immunity

**DOI:** 10.3389/fimmu.2017.00861

**Published:** 2017-07-25

**Authors:** Emmanuel C. Patin, Selinda Jane Orr, Ulrich E. Schaible

**Affiliations:** ^1^Priority Area Infections, Department Cellular Microbiology, Forschungszentrum Borstel, and German Center for Infection Research, TTU-TB, Borstel, Germany; ^2^Division of Infection and Immunity, School of Medicine, Cardiff University, Cardiff, United Kingdom

**Keywords:** macrophage inducible C-type lectin, trehalose dimycolate, anti-inflammatory, phagocytosis, innate immunity

## Abstract

The macrophage-inducible C-type lectin (Mincle) is an innate immune receptor on myeloid cells sensing diverse entities including pathogens and damaged cells. Mincle was first described as a receptor for the mycobacterial cell wall glycolipid, trehalose-6,6′-dimycolate, or cord factor, and the mammalian necrotic cell-derived alarmin histone deacetylase complex unit Sin3-associated protein 130. Upon engagement by its ligands, Mincle induces secretion of innate cytokines and other immune mediators modulating inflammation and immunity. Since its discovery more than 25 years ago, the understanding of Mincle’s immune function has made significant advances in recent years. In addition to mediating immune responses to infectious agents, Mincle has been linked to promote tumor progression, autoimmunity, and sterile inflammation; however, further studies are required to completely unravel the complex role of Mincle in these distinct host responses. In this review, we discuss recent findings on Mincle’s biology with an emphasis on its diverse functions in immunity.

## Introduction

Upon initial encounter with infectious invaders or cellular stress conditions, the host defense immune system has to rapidly recognize pathogens or danger signals as potentially harmful. For this purpose, innate immune cells, such as macrophages, dendritic cells (DCs), and neutrophils (PMN) use a limited number of pattern recognition receptors (PRRs), including toll-like receptors (TLRs), nucleotide-binding oligomerization domain-like receptors, and C-type lectin receptors (CLRs), which activate immediate anti-microbial effectors or other defense mechanisms. These PRRs can sense conserved structural motifs of both microbial and endogenous danger signals, i.e., pathogen-associated molecular patterns (PAMPs) and damage-associated molecular patterns (DAMPs), respectively (1, 2). The macrophage-inducible C-type lectin (Mincle) belongs to the CLR family, along with Dectin-1 (CLEC7A), Dectin-2 (CLEC6A), macrophage C-type lectin (MCL) (CLEC4D), macrophage mannose receptor (MMR, CD206) as well as dendritic cell-specific intercellular adhesion molecule-3 grabbing non-integrin (DC-SIGN, CD209) (1–3). Initial reports demonstrated that Mincle, similar to Dectin-2 and MCL, associates with the Fc receptor gamma (FcγR) signaling chain, which contains an immunoreceptor tyrosine-based activation motif (ITAM). Phosphorylation of the associated FcγR ITAM leads to recruitment of spleen tyrosine kinase (Syk), which activates Card9-Bcl10-Malt1-mediated NF-κB signaling pathway and subsequent expression of pro-inflammatory cytokines (2, 4, 5).

Over recent years, Mincle gained growing interest as shown by an exponentially increasing number of publications on its complex biology. Excellent reviews have already emphasized the beneficial role of Mincle during infection in triggering pro-inflammatory responses as well as its recognition of the mycobacterial ligand, trehalose-6,6′-dimycolate (TDM or cord factor), and the putative utilization of synthetic homologs as better adjuvants for vaccination (5–9). Based on recent studies, we discuss herein Mincle’s expression patterns, rapidly expanding ligand diversity, and interactions with ligand relevant for adjuvant design as well as novel facets of Mincle’s functions in immune modulation, phagocytosis, cancer surveillance, and autoimmunity.

## Mincle’s Fine-Tuned Expression in Immune Cells

Macrophage inducible C-type lectin was first identified in the late 1990s by Matsumoto and colleagues who observed increased expression of the Mincle encoding CLEC4E gene in macrophages following stimulation with inflammatory agents such as TLR agonists, including LPS, but also pro-inflammatory cytokines such as IFNγ, TNF-α, and IL-6 (10). Importantly, the increase in Mincle expression was dependent on the nuclear factor NF-IL6 and MyD88 (10–12). To date, Mincle has been shown to be mainly expressed on myeloid cells, including macrophages, monocytes, DCs, and PMN (5, 10, 13–15) although one report also described Mincle expression on human B cell subsets (16). Induction of Mincle expression was observed upon infection with mycobacteria (17, 18), *Leishmania major* (*L. major*) (19) and treatment with the *Agrocybe aegerita* lectin (AAL) (20). In addition to TLR-mediated signals, induction of Mincle expression was also shown to depend on MCL (CLEC4D). In contrast to Mincle, which is weakly expressed in the absence of inflammatory stimuli, myeloid cells were shown to express MCL constitutively in macrophages and DCs (21), whereas MyD88 was critical for its surface translocation (12). Notably, MCL and Mincle share ligands such as TDM, which strongly suggests synergy between both receptors and a two-step control of the downstream signaling pathway (21). Recent investigations indicate that MCL and Mincle form a complex heterodimer that is translocated to the cell surface during microbial challenge (12, 22, 23). One could speculate that a certain threshold of MCL ligands further enhances sensing of those triggers by increasing Mincle expression to better regulate host responses. Of note, Schoenen and co-workers recently demonstrated the critical function of the early growth response transcription factor C/EBPβ for the expression of Mincle in macrophages, which failed to respond to TDB/TDM stimulation in the absence of this factor (11).

Little is known about the molecular mechanisms involved in the negative modulation of Mincle expression. However, one group recently showed that a cocktail of GM-CSF and IL-4 can downregulate Mincle mRNA transcripts in human DCs (24). Follow-up investigations from the same group extended previous observations by showing that IL-4 inhibits Mincle expression during *in vitro* differentiation of murine bone marrow-derived macrophages and DCs in a STAT-6-dependent manner (25). Interestingly, stimulation by the TLR4 ligand, LPS, counteracts IL-4-mediated suppression of Mincle expression in myeloid cells.

## Mincle, a Promiscuous Sensor of Diverse Stimuli

Diverse structures originating from both the mammalian host as well as microbes have been identified as ligands for Mincle. Mincle thereby acts as a PRR for infectious as well as endogenous inflammatory conditions. All currently described putative ligands of Mincle are listed in Table 1. However, some of these ligands most likely require further biochemical evaluation in order to pivotally clarify whether these are true Mincle ligands or whether their observed reactivities were due to minor contaminations with other PAMPs, a lesson learned from studies on putative TLR2 and TLR4 ligands (26, 27).

**Table 1:** Microbial or endogenous ligands of macrophage inducible C-type lectin (Mincle).

Mincle ligand	Origin	Selected publications
Sin3-associated protein 130	Damaged and necrotic cells	Yamasaki et al. (4)
β-glucosylceramide	Damaged cells	Nagata et al. (31).
Cholesterol crystals	Bovine liver	Kiyotake et al. (32)
Cholesterol sulfate	Skin epithelium	Kostarnoy et al. (33)
Trehalose-6,6′-dimycolate	Mycobacteria	Ishikawa et al. (47); Schoenen et al. (48)
Glycerol monomycolate (GroMM)	Mycobacteria	Hattori et al. (49)
β-gentiobiosyl diacylglycerides	*M. tuberculosis*	Richardson et al. (50)
Glucosyl-diacylglycerol	*Streptococcus pneumoniae*	Behler-Janbeck et al. (44)
α-glucosyl diglyceride	*Lactobacillus plantarum*	Shah et al. (46)
Brartemicin	*Nonomuraea* spp.	Jacobsen et al. (92)
*Agrocybe aegerita* lectin	*Agrocybe aegerita*	Zhang et al. (20)

Macrophage-inducible C-type lectin has been shown to sense mammalian cell components or DAMPs such as the histone deacetlyase complex unit SAP130 alarmin, a protein released from damaged and necrotic cells (4, 28). Recombinant SAP130 triggers pro-inflammatory cytokine secretion such as MIP-2 from peritoneal macrophages in a FcγR chain-dependent manner (4).The relevant role of Mincle for endogenous inflammatory conditions is corroborated by concomitant upregulation of Mincle, SAP130, and phospho-Syk expression in ischemia (29), pancreatic ductal adenocarcinoma (PDA) (28), as well as ethanol-induced liver injury (30). More recently, Nagata and coworkers identified β-glucosylceramide, an ubiquitous intracellular metabolite also released from damaged cells, as a ligand for Mincle (31). Accordingly, this endogenous component was able to induce immunostimulatory functions in myeloid cells, which was abbrogated in the absence of Mincle. In addition, crystalline cholesterol present in atheriosclerotic plaques, inflammatory foci associated with macrophage infiltrates, was shown to bind to human, but not mouse Mincle, thereby inducing pro-inflammatory cytokines (32). Furthermore, it was recently reported that Mincle could sense cholesterol sulfate in a sterile skin inflammation model in mice (33).

In addition to binding DAMPs, Mincle also interacts with PAMPs from various microbes. Initial studies reported Mincle as the receptor for the mycobacterial cell wall glycolipid TDM (6). Consequently, Mincle was also found to be involved in protective immunity to both, *Mycobacterium bovis* bacillus Calmette–Guérin (BCG) and *M. tuberculosis* Erdman. However, an independent study demonstrated that Mincle is dispensable for the control of *M. tuberculosis* H37Rv in mice (15, 17, 34). The different routes of administration, i.e., systemic versus airway, might explain the discrepancies between those reports. Differences in TDM content or localization between the mycobacterial strains used in those studies may also affect Mincle sensing and subsequent innate responses. In addition to Mincle, TDM can also engage MCL (21). Recent investigations have revealed a critical function of MCL in *M. tuberculosis* infection, which was demonstrated by enhanced mortality rates and aggravated inflammation in MCL-deficient mice when compared to their WT counterparts (35).

Macrophage inducible C-type lectin has also been described as important player in immunity to fungal pathogens such as *Candida* (*C*.) *albicans* and *Malassezia* species (36, 37). However, the fungal cell wall component acting as a ligand for Mincle has still not yet been fully identified although two distinct glycolipids isolated from *Malassezia* were shown to bind Mincle (38) (Table 2).

**Table 2:** Future questions on macrophage inducible C-type lectin (Mincle)’s immune functions.

1.	Which are the physicochemical properties of *Helicobacter pylori, Fonsecaea monophora, Leishmania major, Candida albicans*, and *Pneumocystis carinii* ligands recognized by Mincle?
2.	Can the promiscuity of Mincle’s ligand interactions be explained by structural analogies between ligands?
3.	Is Mincle recruited to the phagocytic synapse together with other receptors?
4.	Are Mincle SNPs associated with increased susceptibility to cancers and autoimmune diseases?
5.	Is Mincle involved in autoimmunity in the absence of respective ligands during the induction phase?
6.	Is targeting Mincle a strategy to cure infections, cancers, and autoimmune diseases?
7.	Is Mincle playing a role in sterile inflammation?

Over recent years, the list of pathogens recognized by Mincle has expanded tremendously, including *Streptococcus* (*S*.) *pneumoniae, Fonsecaea* (*F*.) *monophora, Helicobacter* (*H*.) *pylori, L. major, Pneumocystis carinii* as well as different *Corynebacterium* strains (19, 39–43). From many of these pathogens, the specific ligands that bind Mincle are yet unidentified. However, one group recently reported Mincle as receptor for glucosyl-diacylglycerol from *S. pneumoniae*, and this interaction was demonstrated to determine the outcome of experimental pneumococcus infection in mice (44). It was also shown that a galectin isolated from *Agrocybe aegerita* mushroom could act as ligand for Mincle (45). In addition, Mincle has also been described as a PRR for cyclopropane-fatty acid α-glucosyl diglyceride, a product of the commensal probiotic *Lactobacillus (L.) plantarum* (46). Therefore, Mincle is not only involved in sensing infectious or endo-inflammatory conditions but may also act as sensor for changes in microbiota community compositions and may, therefore, contribute to microbiota associated immundomulation. Taken together, Mincle recognizes a heterogeneous array of ligands most of them not identified yet. Whether Mincle’s promiscuous ligand interactions are based on common structural analogies is awaiting further studies (Table 2).

As supported by numerous investigations, TDM is the best Mincle ligand characterized. Interaction between Mincle and TDM triggers pro-inflammatory cytokine secretion in macrophages (11, 47). Consequently, Mincle-deficient mice showed impaired production of inflammatory cytokines and chemokines upon infection with *M. bovis* BCG or exposure to oil-in-water emulsion containing TDM (15, 48). *In vitro*, deletion of Mincle impairs TDM-mediated induction of pro-inflammatory cytokine release by murine macrophages (47). Another mycolic acid derivatized compound, glycerol monomycolate (GroMM), derived from mycobacteria was recently identified as a Mincle ligand (49). Interestingly, further structurally unrelated *M. tuberculosis* metabolites, β-gentiobiosyl diacylglycerides, are sensed by murine but not human Mincle. However, a synthetic truncated β-glucosyl diglyceride emerged as a superior agonist of murine as well as human Mincle (50).

To summarize, the list of endogenous and exogenous ligands recognized by Mincle is steadily growing supporting the critical role of this innate immune receptor in detecting various stimuli under stress conditions.

## Mincle Activation: A Promising Path for the Design of New Adjuvants

Macrophage inducible C-type lectin ligands including various DAMPs and PAMPs, but also synthetic ones such as TDB, are promising adjuvants for vaccine therapies (24, 51–53). Complete Freund’s adjuvant (CFA), which was extensively used for optimal immunization in animal models, also contains mycobacterial cell wall glycolipids including TDM but is to inflammatory for usage in humans.

As critical step in designing new adjuvants, the mechanism of ligand recognition by Mincle has been investigated intensively over recent years. The ultimate aim is to design synthetic Mincle ligands, which retain their robust adjuvancy while their exacerbated inflammatory properties are reduced for safer usage.

The structure–function relationships during molecular Mincle–ligand interactions have already been the subject of excellent reviews (5, 54, 55). To date, structural studies have identified various ligand-binding sites in Mincle (56–59). A glycolipid ligand has been shown to interact with Mincle *via* three binding sites: (I) a primary canonical Ca^2+^ based C-type binding site with similarity to sites in other C-type lectins; (II) a proximal secondary binding site for a glucose residue in the trehalose headgroup; (III) a shallow hydrophobic region, which can bind acyl chains and is in close proximity to the putative sugar binding site in a similar manner as in MCL (56, 59). The latter two binding sites likely act in concert to interact with both, the hydrophilic sugar head group as well as the fatty acid chains of glycolipid ligands, which may modulate affinity and/or specificity. Recently, Feinberg and co-workers demonstrated that the affinity of Mincle for hydrophobic ligands correlates with the number of carbon atoms. This was irrespective of the ligand’s fatty acid side chain structures, i.e., linear, branched, aromatic rings, which could be responsible for a certain level of promiscuity in Mincle’s interactions with hydrophobic ligands. In addition, the structural conformation of Mincle displays a certain degree of flexibility, which might influence the structural properties of the hydrophobic groove and, therefore, ligand binding (58). However, further studies are required to fully understand the structural basis of Mincle’s promiscuity in ligand binding.

Several studies have investigated the potential of synthetic ligands to induce Mincle-mediated immune responses *in vitro* and *in vivo*. Ostrop and co-workers demonstrated strong Mincle-mediated cytokine production from human primary macrophages and DCs in response to TDB/TDM stimulation *in vitro* (24). Synthetic mycolic acids, such as trehalose, glucose, and arabinose monomycolates, were shown to induce production of cytokines and reactive oxygen species as well as surface expression of costimulatory molecules in DCs through Mincle binding (60). Immunization of mice with liposome-based antigen preparations containing synthetic Mincle ligands, including trehalose diesters and monoesters led to robust antigen-specific Th1/Th17 responses (51). Rationally designed Mincle ligands, namely 2-tetradecyloctadecanoic acid and (GlcC14C18) mannose 2-tetradecyloctadecanoic (ManC14C18), have been shown to possess potent immunoprotective activity against *M. tuberculosis* infection (61).

## Mincle Modulates Inflammatory Responses

The role of Mincle as a receptor for pathogenic stimuli, which subsequently triggers innate pro-inflammatory responses, is well established [reviewed in Ref. (5)]. However, recent reports indicate that Mincle rather than purely inducing pro-inflammatory responses is an immune modulator as its engagement also promotes expression of anti-inflammatory cytokines and counter regulates pro-inflammatory signaling pathways (62).

Enhanced secretion of IL-10 has been reported to increase susceptibility of mice to infections by mycobacterial and fungal pathogens (63–67). Moreover, blocking IL-10 production enhanced the protective efficacy of BCG vaccination against *M. tuberculosis* infection (68). Zhang and colleagues demonstrated the ability of neutrophils to secrete IL-10 in response to TDM and concomitant TLR signals (69) and IL-10 production by DCs was also reported upon stimulation with TDB (70). Recently, we revealed the critical role of Mincle and FcγR-Syk signaling pathway for induction of IL-10 secretion *in vitro* and subsequent downregulation of IL-12p40 in response to TDM or *M. bovis* BCG (18). It should be noted that IL-10 secretion as induced by TDM requires a concomitant TLR2 signal, which is provided by whole mycobacteria containing lipoproteins.

Other studies have also revealed an important role for Mincle in the induction of IL-10 expression in BMDMs or BMDCs upon infection with *Malassezia* spp. (37, 38). Moreover, Mincle was shown to induce anti-inflammatory IL-10 responses in human THP-1 cells challenged with *H. pylori* (40). Considering the role of Mincle in regulating IL-10 production, it would be interesting to analyze whether inhibition of IL-10 at the same time of vaccination using TDB as an adjuvant can increase protection to the respective infections. Finally, it remains to be elucidated whether engaging Mincle signaling can trigger expression of other anti-inflammatory cytokines other than IL-10 such as TGF-β and IL-27. However, our preliminary studies failed to reveal production of IL-27 upon TDB stimulation *in vitro* (71).

Besides induction of anti-inflammatory cytokine secretion, Mincle has recently been implicated in the downregulation of pro-inflammatory signals. Investigations from Wevers and colleagues revealed that *F. monophora* engagement of Mincle inhibits Dectin-1-mediated IL-12p35 responses in human DCs (41, 72). Similar results were obtained with TDB, which was shown to interfere with LPS-mediated induction of IL-12p35. Interestingly, *F. monophora*-mediated inhibition of IL-12p35 was abrogated upon treatment of human DCs with wortmannin, a pharmacological inhibitor of phosphoinositide-3-kinase (PI3K). Inhibition of the PI3K effector molecule PKB (or Akt) also blocked Mincle suppression. The authors further concluded that Mincle-mediated activation PI3K/PKB signaling leads to impaired protective Th1 immunity *via* proteasomal degradation of interferon regulatory transcription factor (IRF)-1. Interestingly, another study revealed Mincle-mediated suppression of Dectin-2/FcγR/Card9-induced Th17 response during *F. monophora* infection (73). Indeed, while Dectin-2-deficient mice displayed impaired IL-17 production in lymph nodes of infected mice, this was abrogated in Mincle-deficient animals, thereby revealing a dual function of Mincle and Dectin-2 during fungal infections. More recently, an alternative inhibitory Mincle/FcγR/SH2-domain-containing inositol polyphosphate 5′ phosphatase (SHP-1) signaling pathway was described in *L. major* infection (19). It was demonstrated that Mincle could impair DC activation through SHP-1 recruitment. As a consequence, Iborra and colleagues observed less inflammatory lesions and more robust IFNγ-producing-CD4^+^ T cell responses in the absence of Mincle. Finally, Mincle-deficient mice were less susceptible to experimental Leishmaniasis as shown by decreased parasite burden when compared to their WT counterparts (19).

Other studies have reported Mincle-mediated suppression of TLR2 and TLR4 signaling. Indeed, it was shown that interference with Mincle signaling significantly increased the production of pro-inflammatory cytokines such as TNF-α and IL-6 in splenocytes, BMDCs, and BMDMs upon LPS stimulation. Mincle-deficient mice were likewise more susceptible to an endotoxic shock syndrome-associated pro-inflammatory cytokine storm when compared to their WT counterparts (74). While we observed that TDM-Mincle-mediated IL-10 production in macrophages downregulated TLR2-induced IL-12p40 secretion (18), Mincle-mediated suppression of TLR4-induced cytokine secretion was independent of IL-10. Furthermore, Mincle-deficient splenocytes were shown to express less inhibitory proteins such as SOCS1, A20, and ABIN3, but increased amounts of the TLR4 co-receptor, CD14, in response to LPS. Increased CD14 expression in the absence of Mincle signaling was responsible for enhanced LPS-induced cytokine production (74).

Finally, Lee and co-workers recently revealed a contribution of Mincle to enhanced NOS-2 expression, which involves p38-mediated eIF5A hypusination (45). This mechanism subsequently inhibits the NLRP3 inflammasome and caspase-1-dependent IL-1β secretion by murine macrophages through elevated nitric oxide (NO) production (45). Upon TDM injection, NOS-2-deficient mice developed larger granulomas than wild type ones.

**Figure 1:**
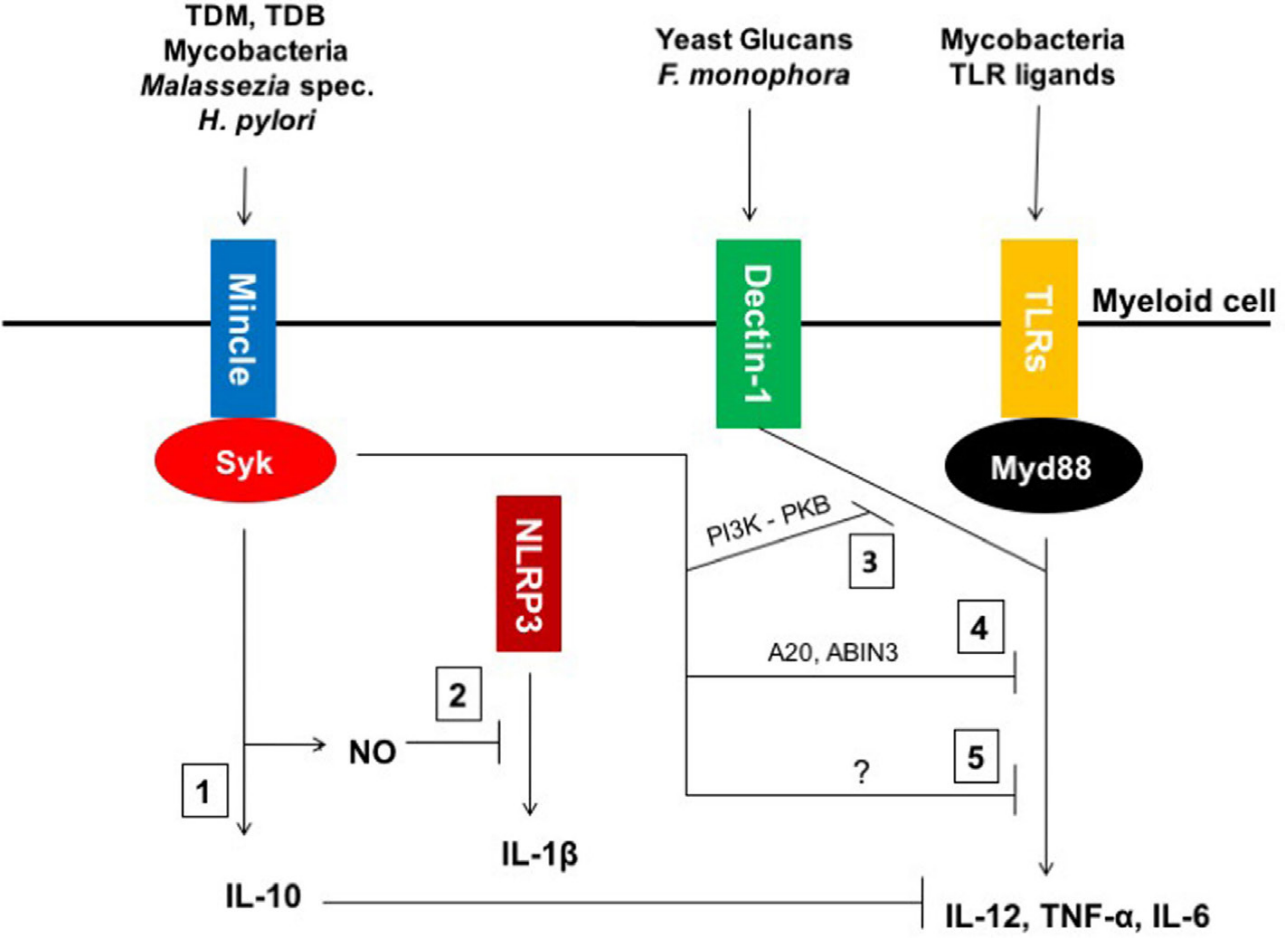
Macrophage inducible C-type lectin (Mincle)-mediated modulation of inflammatory responses. (1) Mincle ligands, such as mycobacterial Trehalose-6,6′-dimycolate (TDM) and its synthetic homolog TDB as well as whole mycobacteria, *Helicobacter pylori* and fungi of the *Malassezia* genus induce IL-10 production by engaging Mincle. (2) Mincle triggers the upregulation of NOS-2 expression, which leads to nitric oxide (NO) production and concomitant inhibition of NLRP3 inflammasome activation and subsequent IL-1β expression in murine macrophages. (3) Mincle impairs *Fonsecaea monophora* Dectin-1-mediated pro-inflammatory cytokine secretion through inhibition of pharmacological inhibitor of phosphoinositide-3-kinase (PI3K)-PKB signaling. (4) Mincle activates both inhibitory intermediates A20 and ABIN3 to downregulate toll-like receptor (TLR)4 signaling. (5) Mincle also inhibits mycobacteria-mediated IL-12 *via* an unknown mechanism.

Thus, as depicted in Figure 1, recent investigations have shown that Mincle can act as immune modulator in different models by either triggering anti-inflammatory responses or downregulating pro-inflammatory signals.

## Is Mincle a Modulator During Phagocytosis?

Phagocytosis is an essential effector mechanism in innate immunity to eliminate pathogens or infected cells. Moreover, in multicellular organisms, phagocytosis is part of the maintenance system to assure tissue homeostasis by clearing necrotic and apoptotic cells (75). Several studies raised the question whether Mincle could be involved in phagocytosis similar to other Syk-coupled CLRs such as Dectin-1 (76, 77). However, this question has not yet been satisfactorily answered.

An early report from Wells and colleagues suggested Mincle as a non-phagocytic receptor in *C. albicans* infection (36). Although the authors described significant recruitment of Mincle to yeast-containing phagosomes in murine macrophages, Mincle was not required for fungal uptake although confocal microscopy analysis demonstrated localization of Mincle around *C. albicans* at the newly formed macrophage phagocytic cup (36). However, neither treatment of macrophages with an anti-Mincle blocking antibody nor using Mincle-deficient macrophages altered the number of phagocytosed yeasts when compared with untreated or control macrophages, respectively (36). From those observations, the authors concluded that the role of Mincle was restricted to sensing *C. albicans* concomitantly with the phagocytic process. An exclusive sensing function of Mincle was also observed in the *S. pneumoniae*–macrophage interaction. Using a recombinant Mincle–Fc fusion protein, Rabes et al. reported that Mincle was capable of binding heat-inactivated *S. pneumoniae* but was dispensable for pneumococcus internalization by professional phagocytes, including macrophages and neutrophils, and for inducing either pro-inflammatory cytokine secretion (39).

**Figure 2:**
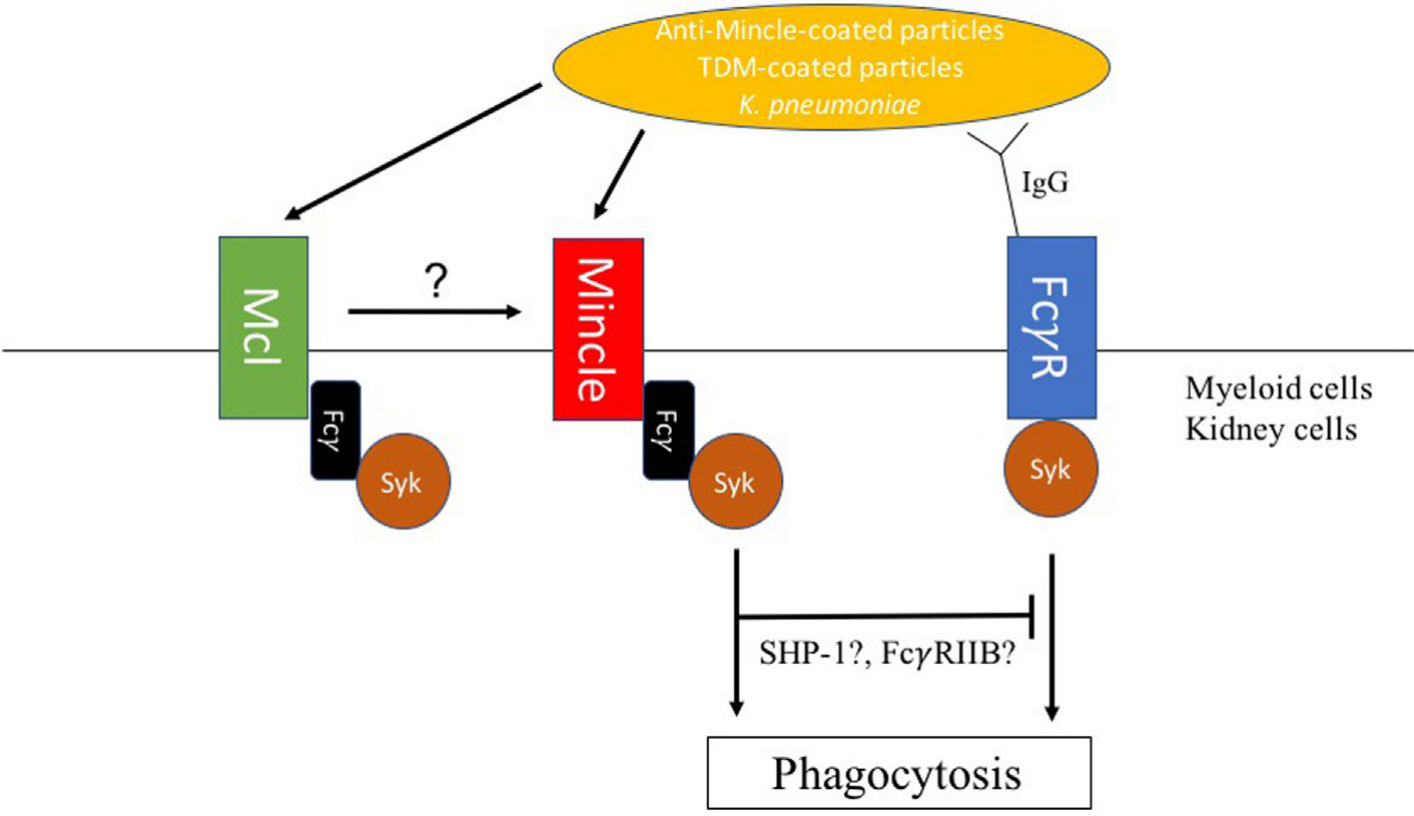
Macrophage inducible C-type lectin (Mincle)-mediated modulation of phagosome biogenesis. (1) Mincle mediates uptake of *Klebsiella pneumoniae* in neutrophils. (2) Mincle and macrophage C-type lectin (MCL) synergize in phagocytosis of anti-Mincle-coated particles by human embryonic HEK-293 cells. (3) Upon FcγR-mediated phagocytosis, trehalose-6,6′-dimycolate (TDM) decelerates phagosome maturation through Mincle signaling. TDM-mediated deceleration of IgG-coated bead particles phagosome maturation required both SHP-1 and FcγRIIB indicating a synergistic inhibitory signal downstream of Mincle, which is, however, associated with the phagocytic process.

In contrast, Mincle was implicated as a receptor for non-opsonic phagocytosis of *Klebsiella pneumoniae* (78). Indeed, lower phagocytosis rates of non-opsonized *K. pneumoniae* were observed by flow cytometry in Mincle-deficient neutrophils when compared with WT control neutrophils (Figure 2). Lobato-Pascual and colleagues investigated the role of Mincle in phagocytosis of anti-Mincle coated particles by 293T cells transfected with Mincle (Figure 2) (23). Phagocytosis of anti-Mincle-coated beads was detected in cells expressing Mincle, but interestingly, phagocytosis was synergistically increased in cells expressing both MCL and ITAM-containing FcγR in addition to Mincle. Those results showed the importance of close association between Mincle, MCL, and FcγR for optimal uptake of anti-Mincle-coated particles. Importantly, MCL has also been reported to be involved in the uptake of mycobacteria by neutrophils, which was taken as explanation for the higher susceptibility to experimental tuberculosis of MCL-deficient mice (12).

Cell wall glycolipids have been shown to interfere with phagocytosis through modulation of phagosome maturation following PRR surface recognition. For instance, mannose-capped lipoarabinomannan (ManLAM) engaging the MMR during phagocytosis was suggested as a mechanism to direct and maintain ManLAM-coated particle in early endosomal phagosomes in human macrophages (79). Similarly, higher-order phosphatidyl-inositol mannosides (PIMs) such as PIM_6_ or glycopeptidolipids were shown to inhibit phagosome maturation by binding to the MMR (80, 81). Importantly, TDM has been proposed as one of the main mycobacterial glycolipids responsible for interference with phagosome maturation. Indrigo and colleagues showed that reconstitution of delipidated mycobacteria with purified TDM restored the ability of the bacteria to inhibit trafficking of phagosomes to lysosomes (82). We and others described delayed maturation of phagosomes containing TDM-coated beads when compared with uncoated beads (82, 83). Phagosomes containing TDM-coated beads retained early endosomal characteristics for a longer time period than phagosomes containing beads coated with the TDM precursor, trehalose monomycolate, or non-related lipids. This indicates that the fatty acid chain is a determinant for TDM-mediated interference with phagosome maturation. Notably, IFN-γ-activation of macrophages overcomes TDM-induced delay of phagosome maturation through NO-mediated alteration of TDM’s hydroxyl residues (83). Those observations suggested a potential role of Mincle during phagocytosis of TDM-coated particles. However, a recent study utilizing several fluorescent trehalose glycolipid reporter systems showed that Mincle was not involved in the uptake of TDM or TDM-coated particles (84), which reinforced the hypothesis of a non-phagocytic role of Mincle. However, Mincle was involved in decelerating bead phagosome maturation, when TDM beads were additionally opsonized with specific IgG (Figure 2). Therefore, parallel engagement of Mincle interferes with FcγR receptor initiated phagosome biogenesis (85). A recent study by Iborra and colleagues demonstrated that Mincle employs a SHP-1-coupling cellular signaling pathway to dampen adaptive immunity to *L. major* infection (19). Interestingly, our investigations showed that TDM-mediated deceleration of phagosome maturation also required SHP-1 suggesting an inhibitory signal downstream of Mincle during the phagocytic process (Figure 2). TDM-mediated inhibition of IgG bead phagosome maturation additionally required the inhibitory FcγRIIB, which suggests collaboration between Mincle and FcγRIIB receptor signaling pathways in modulating phagocytosis of IgG-opsonized TDM-containing particles (Figure 2) (85). Corroborating this notion, association between Dectin-1 and FcγRIIB was previously shown to be required for the inhibition of neutrophil function by IgG1 immune complexes (86). In the context of FcγRIIB’s role in delaying IgG-TDM bead phagosome maturation, it should be noted that mice lacking FcγRIIB were less susceptible to *M. tuberculosis* infection than wild-type ones (87). However, whether Mincle can also modulate phagosome biogenesis upon phagocytosis of particles, which expose other ligands on their surface requires further studies. As mycobacteria triggers strong antibody responses (88), Mincle function upon BCG vaccination would need to be reassessed.

## Mincle as an Endogenous Sensor in Cancer and Autoimmunity

Macrophage inducible C-type lectin can be exploited as a target for TDB adjuvant to boost vaccine efficacy but also for antitumor immunotherapies (89). However, recent studies also indicate a “natural” role of Mincle in sensing tumors and promoting their progression.

Pioneering studies by Roperto and colleagues revealed increased Mincle expression in bovine urothelial tumor cells when compared to healthy individuals (90). The authors concluded that upregulation of Mincle in bladder cancer cells can influence interactions between tumor and immune cells. The anti-TB vaccine strain BCG is successfully used clinically as an immunotherapy against bladder cancer. The underlaying immune mechanisms are not well understood yet, but BCG derived TDM might be involved in this mechanism. The natural trehalose-derived metabolite, brartemicin, a new pharmacological inhibitor of murine colon carcinoma cells has been also described as a Mincle ligand (91, 92). Furthermore, the likely Mincle ligand AAL was described to exert significant inhibitory activities toward various murine and human tumor cell lines such as mouse sarcoma S-180 and HeLa, respectively (20, 93).

A recent study demonstrated a detrimental role of Mincle in pancreatic tumorogenesis in a model of PDA in mice (28). Seifert and co-workers revealed that Mincle expression in tumor-infiltrating myeloid-derived suppressor cells, macrophages, and DCs can promote necrosome-induced accelerated oncogenesis through ligation of the cellular alarmin SAP130. Notably, pancreatic oncogenesis was decelerated in Mincle-deficient mice, and treatment of mice with TDB can drive tumorogenesis, thereby confirming the deleterious effect of Mincle in PDA.

Besides promoting tumor progression, Mincle also seems to be detrimentally involved in exacerbation of certain autoimmune diseases. In T cell-mediated human autoimmune hepatitis as well as its experimental murine counterpart, SAP130 was strongly enhanced and hepatic innate inflammatory cells overexpressed Mincle triggering exacerbated inflammation, whereas interference with Mincle signaling protected against autoimmune hepatitis (94). Experimental autoimmune uveoretinitis (EAU) develops in mice immunized with the endogenous retinal protein interphoto-receptor retinoid binding protein in CFA. Consequently, mice deficient for Mincle but no other C-type lectins were protected against EAU. This observation indicates that Mincle and its Syk/Card9 signaling pathway can promote the development of experimentally induced autoimmune reactions (95). However, it should be noted that CFA, which is frequently used for immunization with autoantigens in order to break tolerance, contains mycobacterial cell wall constituents including TDM. Therefore, it is questionable whether Mincle is involved in triggering autoimmunity under natural conditions when TDM or other Mincle ligands are absent.

## Concluding Remarks

Over the last decade, great progress has been achieved to understand the role of Mincle in immunity. Recent investigations have demonstrated that immune activation using rationally designed synthetic Mincle ligands represents an interesting strategy to shape immune responses toward robust protection against infectious diseases. However, originally categorized as a pro-inflammatory receptor involved in sensing pathogens and necrotic cells, Mincle is now also considered as important regulatory element in inflammation by promoting anti-inflammatory cytokines and subsequent downregulating pro-inflammatory responses. Moreover, Mincle can also determine signaling events controlling phagocytosis and downstream phagosome maturation. Thereby, Mincle can be exploited by mycobacteria through their virulence-associated glycolipid, TDM, to decelerate phagosome maturation. The multifunctional aspect of Mincle in immune responses becomes even more evident by recent observations that it can promote progression of certain tumors, autoimmune reactions, as well as sterile inflammation. The detrimental role of Mincle in these pathological conditions opens up new possibilities of targeting Mincle signaling as potential immune modulatory therapies.

## Author Contributions

EP, US, and SO participated in conceptualization, writing, review, and editing of the manuscript. US and SO contributed to funding acquisition.

## Conflict of Interest Statement

The authors declare that the research was conducted in the absence of any commercial or financial relationships that could be construed as a potential conflict of interest.
